# Influencing factors of fertility concerns in cancer patients of childbearing age: a systematic review and meta-analysis

**DOI:** 10.3389/fonc.2026.1787347

**Published:** 2026-05-18

**Authors:** Yaxin Wang, Renmei Yang, Zhaoli Zhang

**Affiliations:** 1 Department of Thoracic Tumor Center, Chongqing University Cancer Hospital, Chongqing, China; 2 Nursing Department, Chongqing University Cancer Hospital, Chongqing, China

**Keywords:** associated factors, cancer, fertility concerns, meta-analysis, systematic review

## Abstract

**Purpose:**

This study systematically analyzes factors influencing fertility concerns among cancer patients to inform clinical practice.

**Methods:**

We conducted a comprehensive search of PubMed, Web of Science, Embase, Cochrane Library, and Scopus from inception through August 2024 for studies examining factors associated with fertility concerns in cancer patients. Two researchers independently performed literature screening, data extraction, and quality assessment. Meta-analyses were conducted using RevMan 5.4 and Stata 17.0.

**Results:**

A total of 13 articles were included, including 11 cross-sectional studies and 2 longitudinal studies, including 3282 patients. which showed that depression (*OR* = 1.30, 95%*CI* = 1.14-1.49), higher education level (*OR* = 1.74, 95%*CI* = 1.11-2.73), fertility intentions (*OR* = 5.96, 95%*CI* = 1.35-26.31), having more than one child (*OR* = 0.32, 95%*CI* = 0.23-0.41), being married (*OR* = 0.44, 95%*CI* = 0.26-0.75), full-time employment (*OR* = 1.41, 95%*CI* = 1.03-1.93), endocrine therapy (*OR* = 1.31, 95%*CI* = 1.07-1.62) and reproductive counseling (*OR* = 1.21, 95%*CI=*1.01-1.45) were factors associated with fertility concerns in cancer patients.

**Conclusions:**

This study provides the first quantitative evidence that endocrine therapy and prior fertility counseling are significantly associated with elevated fertility concerns, highlighting potential clinical paradoxes and the complex interplay between medical intervention and psychological distress.

**Systematic review registration:**

https://www.crd.york.ac.uk/prospero/, identifier CRD42024582238.

## 1 Introduction

In 2020, there were 19.29 million new cancer cases worldwide, with 4.57 million occurring in China. Notably, 1.79 million of these cases affected young and middle-aged individuals, reflecting an increasing trend toward younger-onset cancer (1). While advancements in cancer screening, medical technology, and chemoradiotherapy regimens have significantly improved patient survival rates (2), these developments have also led to a rise in fertility intentions among survivors. Furthermore, China’s implementation of the two-child and three-child policies has intensified the reproductive needs of cancer patients, particularly those diagnosed before completing their family planning (3). Studies indicate that nearly half of young cancer patients experience fertility concerns during treatment, including fears of shortened lifespan, genetic cancer risks in offspring, and treatment-induced infertility (4). Fertility concern are defined as an individual’s anxiety regarding reproductive capacity and child-rearing, encompassing personal health, offspring health, and parenting ability (5). For this review, we conceptualize “fertility concerns” as a specific multidimensional construct centered on the emotional and cognitive distress related to one’s ability to procreate and parent biological children after a cancer diagnosis. This distress typically encompasses anxieties about (1): the perceived threat of treatment-induced infertility (2); the health and genetic risks to potential offspring; and (3) the impact of cancer and its sequelae on future parenting capacity. Fertility concerns may impose a greater psychological burden than the cancer diagnosis itself, as these concerns often persist longitudinally throughout the reproductive lifespan. Such enduring anxieties can significantly influence therapeutic decision-making, compromise mental wellbeing, and ultimately diminish overall quality of life in cancer patients (6). A study reported that 51% of reproductive-age patients continue to experience fertility concerns (7). To address these issues, the American Society of Clinical Oncology (ASCO) Fertility Preservation Guidelines recommend early discussions between healthcare providers and patients regarding infertility risks and fertility preservation options (8). However, research on fertility concerns among cancer patients in China remains in its nascent stages, with insufficient clinical attention, limited implementation of fertility preservation technologies, and a lack of targeted intervention strategies. Thus, understanding the associated with fertility concerns in this population is critical for mitigating its impact.

Although numerous studies have explored the determinants of fertility concerns in cancer patients, variations in sample sizes and regional focus have led to inconsistent findings, with a notable absence of quantitative synthesis. To address this gap, this study adheres to the PRISMA (Preferred Reporting Items for Systematic Reviews and Meta-Analyses) guidelines (9). Therefore, this study aims to conduct a comprehensive systematic review and meta-analysis to: (1) quantify the strength of associations between key modifiable (e.g., depression, counseling) and non-modifiable (e.g., parity) factors and fertility concerns; and (2) specifically investigate under-researched clinical factors such as endocrine therapy, providing novel insights to guide targeted supportive care strategies.

## 2 Materials and methods

### 2.1 Literature search strategy

A comprehensive literature search was conducted across multiple databases, including PubMed, Embase, Cochrane Library, Web of Science, and Scopus, covering publications from inception to August 2024. To ensure an exhaustive retrieval of relevant studies, we additionally performed backward reference tracing of the included articles and conducted a systematic search of gray literature sources (including ProQuest Dissertations & Theses Global and major conference proceedings) to minimize publication bias and enhance the comprehensiveness of our evidence synthesis. The search employed the following Medical Subject Headings (MeSH) terms and keywords: Neoplasm-related terms: neoplasms, cancer, malignant neoplasm, tumor. Fertility concern terms: reproductive anxiety, fertility concerns, reproductive worries, fertility worries, fertility anxiety, fertility concerns, childbearing concerns. We developed a comprehensive search strategy in PubMed, as detailed in **Table 1**.

**Table 1:** Search strategy for PubMed.

Search #	Search term
#1	“neoplasms”[Mesh] OR Cancer[Title/Abstract] OR Tumor[Title/Abstract] OR malignan*[Title/Abstract]
#2	(fertility worries[Title/Abstract]) OR (fertility concern[Title/Abstract]) OR (reproductive concern[Title/Abstract]) OR (reproductive worries[Title/Abstract]) OR (pregnant* worries[Title/Abstract]) OR (pregnant* concerns[Title/Abstract])
#3	#1AND#2

To ensure consistency in variable definitions across studies, we implemented the following standardization procedures for meta-analysis:

Education level was dichotomized into “higher education” (university level or above) versus “non-higher education”.

Desire to preserve fertility and intentions to childbearing was defined as a positive response to any question regarding desire for future children.

Marital status was categorized as “married/partnered” versus “single/divorced/widowed”.

Employment status was classified as “full-time employment” versus “other”.

Parity was defined as having “>1 child” versus “≤1 child”.

### 2.2 Inclusion and exclusion criteria for literature selection

#### 2.2.1 Inclusion criteria

1)Study design:Cross-sectional studies or cohort studies; 2)The subjects were patients diagnosed with cancer by pathological examination; 3)Cancer patients aged 18 to 49 years old (all enrolled participants must meet the age criteria, with no mean age substitution); 4)Research tool: a scale assessed the level of fertility concerns in patients; 5)Reported odds ratios (*OR*) with 95% confidence intervals (*CI*) for factors associated with fertility concerns, either directly or derivable from provided data.

#### 2.2.2 Exclusion criteria

1) Unavailable Full Text: Studies for which the full text could not be accessed despite exhaustive search efforts; 2) Studies lacking sufficient data for quantitative synthesis (e.g. missing *ORs*, 95% *CIs*, or key statistical measures); 3) Duplicate Publications: Redundant publications of the same study population; only the most comprehensive or recent version was retained; 4) Low Methodological Quality: Studies rated as low quality based on standardized appraisal tools (e.g.Newcastle-Ottawa Scale for cohort studies); 5) Non-English language articles: Only studies published in English were included due to limitations in language translation and data verification.

### 2.3 Article screening and data extraction

Two independent researchers with formal training in evidence-based methods conducted the literature screening and data extraction in accordance with the predefined inclusion and exclusion criteria. The inter-rater reliability for literature screening and data extraction was evaluated using the Cohen’s Kappa coefficient, with a Kappa value of >0.80 indicating excellent consistency between the two researchers. The process was implemented as follows: Both researchers systematically evaluated titles, abstracts, and full texts. Initial screening results were cross-verified to ensure consistency. Discrepancies in study selection and data extraction were first resolved through face-to-face consensus discussion between the two independent researchers. If no agreement was reached after discussion, the discrepancies were submitted to a third senior researcher with more than 10 years of experience in evidence-based medicine and meta-analysis for binding adjudication, and the final decision was recorded in detail. Extracted data elements included: basic study characteristics (first author, publication year, country). Measurement tools (validated scales for fertility concerns). Key outcomes (*ORs* with 95% *CIs* for all reported factors). All data were systematically recorded in predefined Excel spreadsheets.

### 2.4 Quality assessment

Two independent researchers conducted the quality assessment of all included studies using standardized evaluation tools. The assessment process was implemented as follows: for cross-sectional studies, we employed the 11-item evaluation criteria developed by the U.S. Agency for Healthcare Research and Quality (AHRQ). Studies were classified based on their total scores: high quality: 8–11 points, moderate quality: 4–7 points, Low quality: 0–3 points. For cohort studies, quality was assessed using the Newcastle-Ottawa Scale (NOS), which evaluates studies across three key domains: selection, comparability, and outcome assessment.

To ensure consistency in quality ratings: all assessments were performed independently by two researchers. Discrepancies in quality assessment were first resolved through face-to-face consensus discussion. Any remaining disagreements were adjudicated by a third senior researcher with more than 10 years of experience in meta-analysis.

### 2.5 Statistical analysis

Statistical analysis was performed using RevMan 5.4 and Stata 17.0. The *I*² statistic was used to evaluate heterogeneity among studies. If *I*² ≤ 50% and *P* ≥ 0.10, the fixed-effect model was used for meta-analysis. If *I*² > 50% and *P* < 0.10, significant inter-study heterogeneity was indicated, and the random-effects model using the DerSimonian-Laird estimator was used for meta-analysis. For all analyses, we preferentially extracted and pooled adjusted odds ratios (ORs) when available. If only unadjusted ORs were provided, these were used for synthesis. Studies with zero events in both groups were excluded from meta-analysis, while studies with zero events in one group were handled by adding a continuity correction of 0.5. Quality assessment scores (AHRQ for cross-sectional studies, NOS for cohort studies) were used to inform the interpretation of findings but did not serve as exclusion criteria. To assess the potential impact of study quality on our results, we conducted the following pre-planned sensitivity analyses: Restricting analysis to high-quality studies (AHRQ score ≥ 8 or NOS score ≥ 7). Performing meta-regression to examine the association between quality scores and effect sizes for outcomes with sufficient studies (≥ 10).

Sensitivity analysis was performed for each associated factor using both fixed-effects and random-effects models. The combined effect size was expressed as OR value with 95% *CI*. Publication bias was assessed using Egger’s test for factors reported in five or more studies, with *P* < 0.05 considered statistically significant. The study has been registered with PROSPERO (ID: CRD42024582238).

### 2.6 Theoretical framework for interpretation

To guide the interpretation of our findings, particularly when confronting complex or counterintuitive associations, we adopted a conceptual framework that emphasizes causal inference and the potential for bias in observational data. This framework specifically alerts us to the possibility of confounding by indication and reverse causality when interpreting cross-sectional relationships.

Confounding by indication occurs when the exposure under study (e.g., receiving fertility counseling) is not randomly assigned but is instead influenced by the underlying characteristics or risk profile of the patients (e.g., their pre-existing level of fertility concern). This can create a spurious association where the outcome (high concern) appears to be linked to the exposure (counseling), while the true driver is the clinical indication for receiving that exposure. Reverse causality describes a situation where it is plausible that the outcome influences the exposure, rather than vice versa.

We applied this lens *post-hoc* to contextualize findings where the direction of effect was ambiguous based on the available data, acknowledging that establishing true causality is impossible for cross-sectional studies and requires prospective longitudinal designs.

## 3 Results

### 3.1 Search process

**Figure 1:**
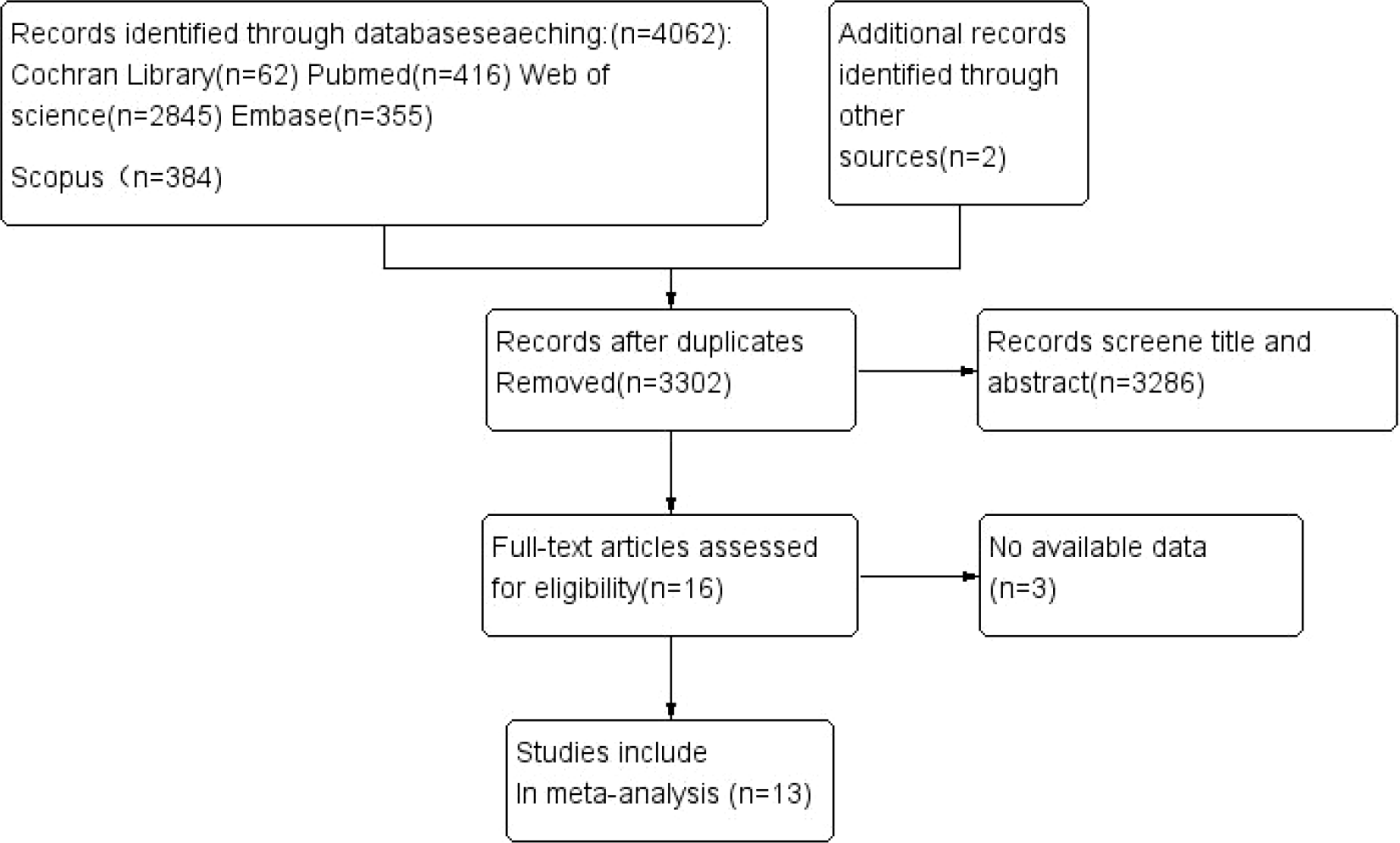
Flow diagram of the literature search.

A total of 4,064 publications were identified through systematic searches across five databases: PubMed, Cochrane Library, Web of Science, Scopus, and Embase. After removing duplicates, 3,302 records remained. Preliminary screening of titles and abstracts yielded 16 potentially eligible studies, of which 13 were ultimately included for final analysis following full-text review. The literature selection process is illustrated in **Figure 1**.

### 3.2 Characteristics of the included studies

This study included a total of 13 articles comprising 3,282 patients, with 11 cross-sectional studies and 2 longitudinal studies. Based on quality assessment, two studies were rated as moderate-quality, while the remaining 11 were high-quality. The basic characteristics, detailed quality scores, and quality evaluation results of the included studies are presented in **Table 2**.

**Table 2:** The characteristics of the included studies.

Author	Year	Country	Sample size	Instrument	Associated factor	Quality assessment
Gorman J R (10)	2015	USA	200	RCAC	A	9
Bártolo A (11)	2020	Portuguesa	104	RCAC	A	6
Jiajia Qiu (12)	2022	China	112	RCAC	C	7
Gorman J (13)	2010	USA	131	RCS	D	8
Young K (14)	2019	USA	747	RCAC	FKM	9
Cherven B (15)	2022	USA	308	RCAC	L	6
Ruddy K J (16)	2014	USA	620	FIS	ACEFGHIJKN	8
Nyeko R (17)	2024	Uganda	110	RCAC	LM	6
He X (18)	2024	China	188	RCAC	CK	9
Ljungman L (19)	2018	Sweden	181	RCAC	CDJK	9
Ruggeri M (20)	2019	Switzerland Italy	297	RCAC	BCEFGHIJKN	8
Wu X (21)	2023	China	150	RCAC	BCD	8
Villarreal-Garza C (4)	2017	Mexico	134	FIS	DEFJ	8

A, depression; B, age; C, education; D, desire to have children; E, >1 child; F, Married; G, full-time job; H, economic; I, genetic; J, endocrine therapy; K, chemotherapy; L, cancer diagnosis; M, reproductive counseling; N, surgery.

### 3.3 Influencing factors and results affecting fertility concerns

#### 3.3.1 Meta-analysis results

The meta-analysis of 13 included studies identified 14 potential associated factors, with factors reported in two or more studies being pooled for analysis. Results revealed several significant associations (*P* < 0.05) with fertility concerns among cancer patients.

Notably, high heterogeneity was observed in several associated factors (e.g., education:

*I*²=83%, fertility intentions: *I*²=83%, being married: *I*²=91%), which may be attributed to differences in study populations (e.g., cancer type, regional cultural background) and operational definitions of factors across studies. Subgroup analysis based on measurement tools showed no significant reduction in heterogeneity, suggesting that unmeasured factors may be the main sources of heterogeneity.

Findings from the meta-analysis indicated that depression, higher educational attainment, fertility intentions, full-time work, endocrine therapy, and previous reproductive counseling were all linked to higher levels of fertility concerns. In contrast, having more than one child and being married were linked to lower levels of such concerns, with all differences reaching statistical significance (*P* < 0.05).

**Figure 2:**
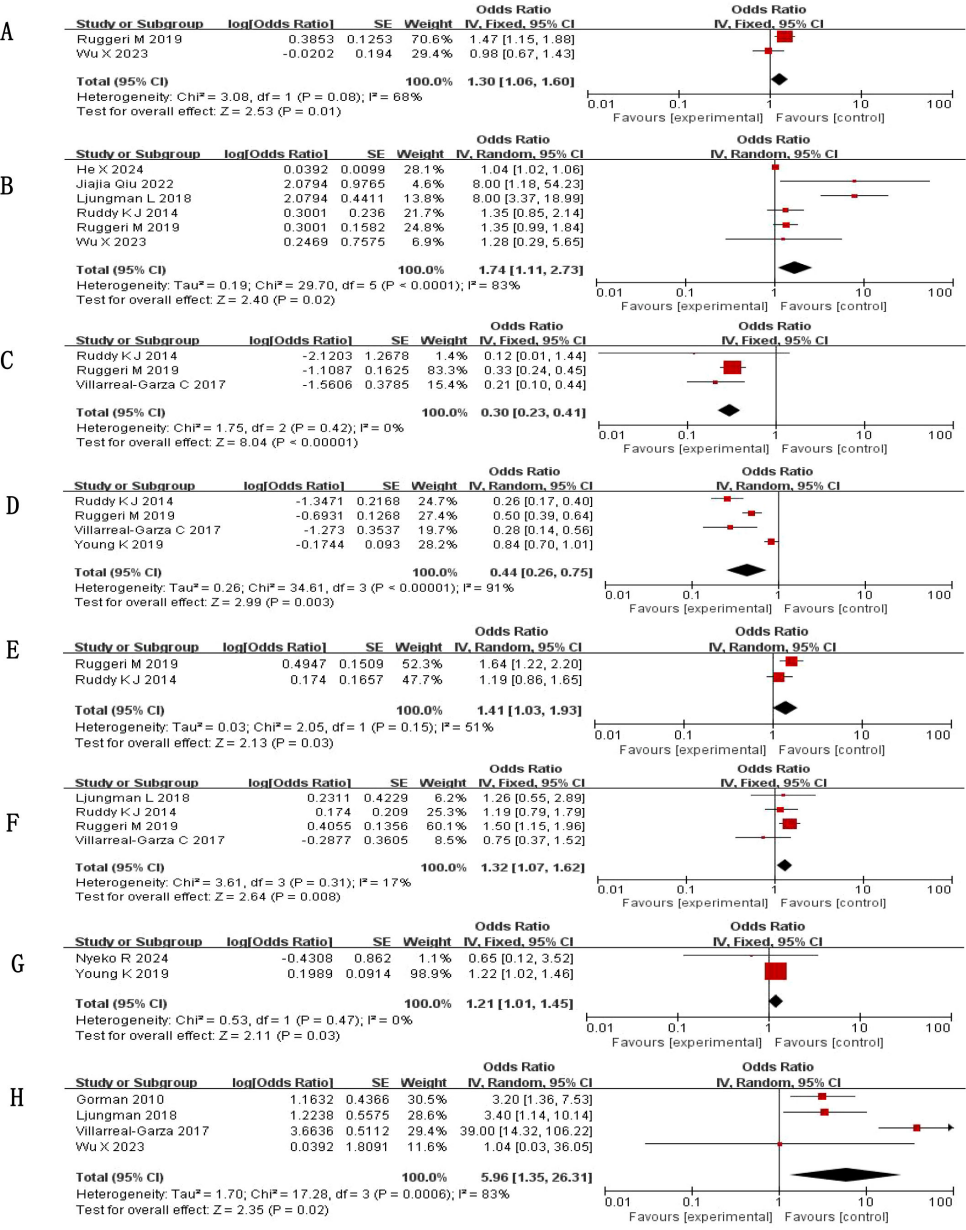
Forest plot of influencing factors in cancer patients. **(A)** depression; **(B)** education; **(C)** >1 child; **(D)** Married; **(E)** full-time job; **(F)** endocrine therapy; **(G)** reproductive counseling; **(H)** Fertility intentions.

The combined effect sizes are shown in **Table 3**, **Figure 2** (Forest plot of statistically significant associated factors for fertility concerns).

**Table 3:** Meta-analysis of associated factors of fertility concerns in cancer patients.

Influencing factors	Combination studies	Heterogeneity test		Model	*OR (*95%*CI*)	*P*
		*I* ^2^	*P*			
depression	3	0%	0.05	Fixed	1.30 [1.14,1.49]	0.0001
age	2	68%	0.08	Random	1.23 [0.83,1.83]	0.3
education	6	83%	<0.0001	Random	1.74 [1.11,2.73]	0.02
Fertility intentions	4	83%	0.0006	Random	5.96 [1.35,26.31]	0.02
>1 child	3	0%	0.42	Fixed	0.32 [0.23,0.41]	<0.00001
Married	4	91%	<0.00001	Random	0.44 [0.26,0.75]	0.003
full-time job	2	51%	0.15	Random	1.41 [1.03,1.93]	0.03
economic	2	71%	0.06	Random	0.94 [0.64,1.37]	0.73
genetic	2	0%	0.86	Fixed	1.10 [0.88,1.37]	0.41
endocrine therapy	4	17%	0.31	Fixed	1.31 [1.07,1.62]	0.008
chemotherapy	5	64%	0.03	Random	1.13 [0.96,1.32]	0.14
cancer diagnosis	2	46%	0.17	Fixed	1.92 [1.01,3.66]	0.05
reproductive counseling	2	0%	0.47	Fixed	1.21 [1.01,1.45]	0.03
surgery	2	85%	0.009	Random	0.70[0.40,1.22]	0.21

Because cancer diagnosis, full-time employment, and reproductive counseling were each evaluated in only two studies, the estimated effect sizes for these variables should be interpreted carefully, as they may lack stability and broad generalizability. Such small sample sizes for synthesis lead to less stable effect estimates that are more vulnerable to the unique characteristics of individual studies; thus, these findings are considered preliminary and require further validation in larger, multicenter cohort analyses with more homogeneous study populations.

#### 3.3.2 Subgroup analysis

**Figure 3:**
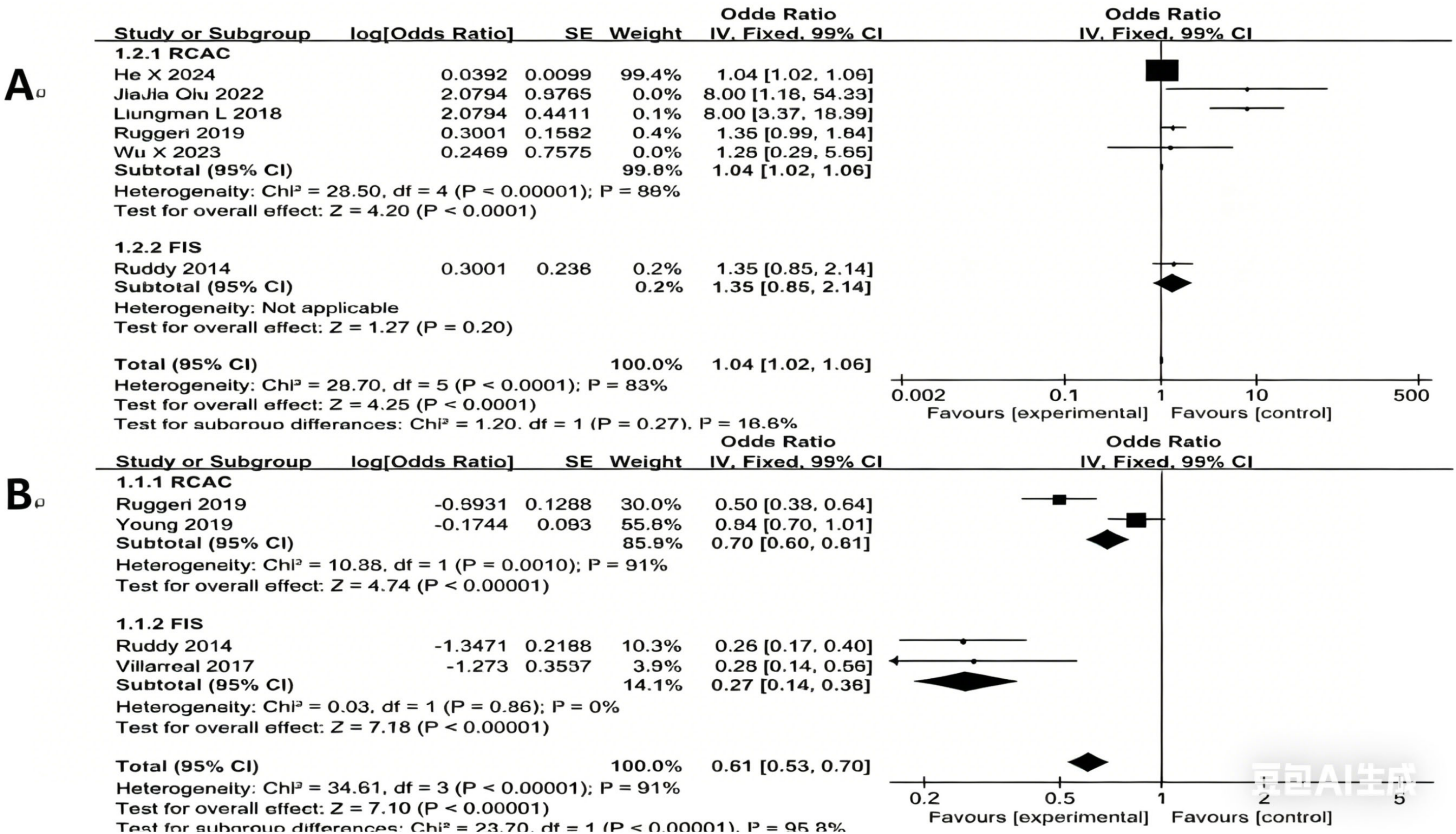
Subgroup analysis of education and married in cancer patients. **(A)** education; **(B)** married.

The results demonstrated that both educational level and married status were significant associated factors that improve the emotional state of the patient, reducing concerns related to fertility, regardless of the assessment tool used (such as RCAC, FIS, or RCS), and this conclusion remained consistent across subgroups. However, the subgroup analysis did not substantially reduce the heterogeneity between studies, suggesting that differences in measurement tools are not the primary source of heterogeneity. The results are presented in **Figure 3** (Subgroup analysis of higher education and being married in cancer patients).

#### 3.3.3 Sensitivity and publication bias analysis

Sensitivity analysis was performed for each in associated factor using both fixed-effects and random-effects models. The consistency between the two models’ results (all *P* > 0.05) demonstrates the robustness and reliability of our findings. To evaluate the influence of study quality, we conducted sensitivity analyses restricting to high-quality studies. The direction and magnitude of effects remained consistent in these analyses, supporting the robustness of our primary findings. For outcomes with sufficient studies, meta-regression did not reveal significant associations between quality scores and effect sizes (all *P* > 0.05).

Publication bias was assessed using Egger’s test for factors reported in five or more studies, with results presented in **Table 4**. For outcomes with fewer than five studies, the power to detect small-study effects is limited. Therefore, we did not conduct formal tests for these outcomes but acknowledge the potential for unpublished negative findings as a limitation. For the factor ‘education’ (Egger’s test *P* = 0.057), we performed a trim-and-fill sensitivity analysis to estimate the potential effect of missing studies. The adjusted estimate remained statistically significant (*OR* = 1.54, 95%*CI* = 1.01-2.36), supporting the robustness of this association.

**Table 4:** Egger test for associated factors.

Associated factors	*t*	*p*
education	2.66	0.057
chemotherapy	2.05	0.133

## 4 Discussion

This meta-analysis synthesized evidence from 13 studies across eight countries to systematically examine factors associated fertility concerns among cancer patients. The analysis identified 14 significant associated factors, including six factors positively associated and factors negatively associated with fertility concerns.

Although the dual independent review process and the application of appropriate quality assessment tools strengthen the methodological rigor, the risk of bias inherent in the included studies remains a concern. For instance, certain cross-sectional studies exhibited selection bias due to non-random sampling methods, while some cohort studies showed potential attrition bias as a result of incomplete follow-up. These biases may have influenced the overall findings of the meta-analysis in several ways: they could lead to an overestimation or underestimation of the true effect size, increase the between-study heterogeneity, and affect the generalizability of the results. Future meta-analyses would benefit from individual participant data or more stringent inclusion criteria to minimize such biases.

Depression demonstrates a strong association with fertility concerns and emerges as a major risk factor for heightened reproductive distress in cancer patients of childbearing age. Following cancer diagnosis, patients frequently undergo surgical interventions or adjuvant therapies that may compromise secondary sexual characteristics and induce various adverse effects, including menstrual disturbances and endocrine dysfunction (22, 23). These physiological changes can significantly impair reproductive capacity and diminish quality of life, creating a bidirectional link between physical reproductive impairment and psychological distress (24). Furthermore, the debilitating nature of cancer coupled with substantial treatment costs often imposes severe financial strain on patients and their families, potentially leading to persistent psychological distress characterized by feelings of inadequacy guilt and hopelessness (25). Notably, previous research has quantified that each unit increase in fertility concern scores corresponds to a 2.423-fold elevation in the risk of developing clinical depression (13).

These findings underscore the critical need for healthcare providers to implement proactive, multi-modal psychological support strategies that address the intertwined reproductive anxiety and depressive symptoms experienced by cancer patients. Beyond targeted counseling for fertility concerns, comprehensive psychosocial interventions should be integrated into routine oncology care to mitigate the cumulative mental distress throughout the cancer journey—from diagnosis and treatment to survivorship. Evidence-based interventions include mindfulness-based stress reduction (MBSR) programs, which have been shown to reduce rumination and anxiety by fostering present-moment awareness and emotional regulation in cancer patients (26).

Supportive psychotherapy, including cognitive-behavioral therapy (CBT) tailored to reproductive distress, can help patients reframe negative cognitions about their fertility potential and develop coping skills to manage treatment-related reproductive fears. Couple-based intimacy enhancement interventions are another valuable approach, as they improve relationship satisfaction and communication between patients and their partners, thereby reducing the sense of isolation that often exacerbates fertility-related anxiety; such interventions have demonstrated promising results in randomized controlled trials for breast cancer survivors experiencing sexual and reproductive distress (27). Additionally, patient empowerment approaches—such as peer support groups with cancer survivors who have navigated fertility preservation and reproductive decision-making—can bolster reproductive confidence by providing real-world insights and emotional validation (28). For patients with severe depressive symptoms comorbid with fertility concerns, collaborative care between oncologists, reproductive specialists, and mental health clinicians is essential, including consideration of pharmacotherapeutic interventions for depression when clinically indicated, in conjunction with fertility-focused psychological support. Collectively, these interventions should be delivered in a longitudinal manner, as fertility concerns and associated mental distress persist across the cancer care continuum and do not resolve with the completion of primary treatment.

Higher educational attainment has been identified as a factor positively associated with fertility anxiety among cancer patients. Research indicates that more highly educated individuals often have greater informational needs regarding their disease and reproductive health, and when these needs remain unmet, they experience elevated anxiety and depressive symptoms (29). Additionally, individuals with advanced education tend to delay childbearing, meaning that at the time of cancer diagnosis, they may not yet have children, thereby exacerbating concerns about future fertility.

Given these findings, healthcare providers should adapt communication strategies according to patients’ educational backgrounds and leverage digital platforms—such as internet-based resources and medical information systems—to deliver accurate, tailored reproductive health education in a safe and accessible manner.

This meta-analysis provides the first quantitative synthesis confirming that endocrine therapy significantly positively associated with fertility concerns in cancer patients of childbearing age. Approximately 75% of breast cancer cases are estrogen receptor-positive, necessitating five to ten years of endocrine treatment (30). While this therapy improves survival outcomes, it also carries risks such as impaired sexual function—including reduced libido and vaginal dryness—which adversely affect quality of life (31).

Llarena et al. (32) surveyed 500 hormone receptor-positive breast cancer patients under age 45 and found that 22% desired future childbearing but were concerned about tamoxifen’s potential impact on fertility. Similarly, a study (19). reported elevated fertility concerns among patients undergoing endocrine therapy. These fertility concerns often influence treatment decisions, highlighting the need for careful patient-provider discussions (33). Future research should explore whether structured, fertility-focused counseling programs, potentially augmented by peer support, can mitigate fertility-related anxiety in patients undergoing endocrine therapy.

A notable gap in existing research on fertility concerns in cancer patients is the limited focus on BRCA1/2 germline mutation carriers, whose distinct reproductive and cancer risks significantly amplify reproductive anxiety. BRCA1/2 mutations confer elevated risks of breast and ovarian cancer, with prophylactic surgeries—bilateral salpingo-oophorectomy (BSO) and mastectomy—as standard risk-reduction strategies (34). While BSO reduces ovarian cancer risk by over 90%, it causes immediate iatrogenic menopause and irreversible infertility in premenopausal patients, making fertility preservation a time-sensitive priority. Even without surgery, BRCA mutations independently accelerate ovarian follicle depletion and premature ovarian insufficiency (POI), reducing reproductive lifespan (35). Adjuvant cancer therapies further compound fertility risks.

Fertility concerns are intensified by complex reproductive decisions, including balancing cancer risk reduction with fertility preservation and concerns about transmitting mutations to offspring (36). Assisted reproductive technologies with preimplantation genetic testing (ART-PGT) offer options for avoiding vertical transmission, but access remains limited, and financial and emotional burdens add to reproductive distress. Current fertility preservation guidelines lack tailored recommendations for BRCA carriers, despite their unique timelines and needs. This gap contributes to unmet informational needs and highlights the necessity of integrated onco-genetic and reproductive care. Future research should quantify fertility concerns in this population and identify modifiable factors that mitigate distress, such as early genetic counseling, personalized fertility preservation, and integrated ART-PGT services. Oncology providers also require specialized training to address fertility concerns proactively at the time of genetic diagnosis, before cancer treatment or prophylactic surgery.

Fertility intentions were strongly associated with greater infertility concerns among cancer patients, suggesting that reproductive attitudes may be linked to post-diagnosis psychological adjustment amid considerable situational pressures. Patients who express a desire for future conception often experience heightened anxiety regarding the potential adverse effects of cancer treatments on fetal viability, gestational health, and maternal well-being (37).

Research indicates that proactive fertility preservation strategies can significantly alleviate patient anxieties about future reproductive outcomes (38).; The American Cancer Society emphasizes the importance of early discussions regarding the reproductive risks associated with cancer therapies and advocates for the availability of fertility preservation options. These include ovarian function protection through pharmacological interventions during chemotherapy, as well as assisted reproductive technologies such as embryo cryopreservation, oocyte freezing, and cryopreservation of ovarian tissue (39). For patients with parenting aspirations, clinicians should proactively provide comprehensive information on fertility preservation methods. Such timely counseling can help mitigate emotional distress by empowering patients to reconcile their reproductive goals with treatment-related health considerations.

The findings indicate that prior fertility counseling was positively associated with higher levels of fertility concerns among cancer patients. Fertility counseling, which typically involves assessing treatment-related fertility risks, recommending personalized preservation options, and supporting informed reproductive decision-making (40), is theoretically intended to alleviate distress. However, our observed association likely reflects confounding by indication, a form of selection bias where patients who are already highly concerned about their fertility or who have strong unmet information needs are precisely those most likely to seek out and receive counseling.

This creates a plausible scenario of reverse causation, where the outcome (elevated fertility concerns) influences the receipt of the exposure (counseling), rather than the exposure causing the outcome. This cross-sectional finding should not be interpreted as counseling causing increased distress, but rather as highlighting a complex associative relationship within the available data. The temporal sequence and causal nature of this association require clarification through prospective, longitudinal studies that measure baseline concern levels prior to the counseling intervention. Should future research confirm potential moderating factors, it would be critical to investigate whether specific aspects of counseling, such as its timing, content, or delivery quality, play a role. For instance, it has been suggested that suboptimal fertility counseling—characterized by inconsistent delivery or inadequate content—might fail to alleviate or could potentially exacerbate pre-existing anxieties (14).

The study findings identify two factors negatively associated with fertility concerns in cancer patients: having more than one child and being married. Patients who have already achieved parenthood experience reduced fertility concerns, as they are less susceptible to familial and societal reproductive expectations (41). This completed family structure diminishes anxieties regarding both personal fertility potential and potential health risks to future offspring.

Furthermore, spousal support plays a crucial protective role. As primary caregivers, spouses provide essential emotional support and facilitate effective communication, which enhances mutual understanding and helps mitigate fertility-related psychological distress (42), enabling couples to openly discuss cancer-related experiences. This supportive dynamic enhances mutual understanding and helps mitigate fertility concerns.

In this study, subgroup analyses were conducted for marital status and educational level according to the measurement instruments used. The results indicated that differences in measurement tools were not the primary source of heterogeneity. The persistence of heterogeneity suggests the potential influence of other unmeasured clinical or methodological factors, such as cultural background, sample age distribution, or differences in treatment phases.

The present study also acknowledges that differences in cancer type (e.g., breast cancer, gynecologic cancer) and treatment phase (e.g., initial treatment, follow-up) may introduce confounding bias, as fertility concerns may vary across different cancer types and treatment stages. However, due to insufficient data in the included studies, subgroup analysis based on cancer type and treatment phase could not be conducted. Future studies should further systematically explore these potential sources of heterogeneity and promote the establishment of a unified core outcome set to enhance comparability across study results and strengthen the robustness of synthesized evidence.

These findings carry meaningful clinical implications for the integrated care of reproductive-;age cancer patients. Routine psychological screening and tailored supportive interventions should be incorporated into standard oncology care to alleviate distress among patients with depressive symptoms. For individuals with higher educational levels, targeted reproductive health education delivered through accessible digital platforms can better address their greater informational needs and reduce uncertainty about future fertility. Patients undergoing endocrine therapy require clear, early communication regarding treatment-related impacts on reproductive function, along with structured, patient-centered oncofertility counseling to support treatment decision-making. For those expressing strong fertility intentions, timely information about evidence-based fertility preservation strategies should be provided to empower informed choice. Given the observed association between prior reproductive counseling and higher fertility concerns, clinical programs should refine the timing, content, and quality of counseling to match individual patient needs rather than amplifying preexisting anxiety. Additional support should also be directed toward strengthening marital and family resources, especially among childless and unmarried patients, to buffer against fertility-related emotional strain. Collectively, these observations underscore the value of a multidisciplinary care model that unites oncologists, fertility specialists, mental health providers, and nursing staff to proactively identify and manage fertility concerns across the entire cancer treatment and survivorship continuum.

## 5 Limitations

This study has several limitations. First, the majority of included studies (11/13) were cross-sectional in design, which precludes the establishment of temporality between associated factors and fertility concerns. It is impossible to determine the sequential relationship between factors and whether reverse causality exists in some associations. Second, the number of studies investigating certain factors was relatively small, and the substantial variation in sample sizes across included studies may affect the robustness of the conclusions for those outcomes. Third, significant heterogeneity existed in some factors included in the meta-analysis, which may reduce the stability of the pooled effect estimates. Subgroup analysis based on measurement tools showed no significant reduction in heterogeneity, suggesting that unmeasured factors (e.g., cancer type, treatment phase, cultural background) may be the main sources of heterogeneity. Fourth, the included studies involved various cancer types and treatment phases, but the lack of stratified data precluded further subgroup analysis, which may lead to confounding bias in the pooled results.

## 6 Conclusion

Through a systematic review and meta-analysis, this study synthesized the evidence on factors associated with fertility concerns among cancer patients of childbearing age. Our quantitative synthesis provided robust evidence that several factors are significantly associated with fertility concerns: depression, higher education level, full-time employment, endocrine therapy, and fertility intentions were found to be positively associated with elevated fertility concerns, whereas having more than one child and being married were negatively associated with fertility concerns. Additionally, factors such as prior reproductive counseling were identified as positively associated with fertility concerns, and their relationships require further validation in prospective longitudinal studies.

The findings underscore that fertility concerns in cancer patients is a complex issue influenced by a constellation of factors. To address this, future research should focus on testing the efficacy of targeted interventions informed by these associations. We hypothesize that a multidisciplinary approach involving reproductive specialists, psychologists, and oncologists could enhance support for these patients. Key hypotheses requiring future validation include whether structured fertility counseling programs, integrated psychosocial screening, and patient navigation services can effectively alleviate fertility concerns and improve the quality of life in this population. The implementation and evaluation of such models in prospective, interventional studies are essential next steps.

## Data Availability

The original contributions presented in the study are included in the article/**Supplementary Material**. Further inquiries can be directed to the corresponding authors.
