# Supplementary material for: Influencing factors of fertility concerns in cancer patients of childbearing age: a systematic review and meta-analysis
Source: Front Oncol. 2026 May 18;16:1787347. doi: 10.3389/fonc.2026.1787347 (PMC13223885; doi:10.3389/fonc.2026.1787347)
Supplement: Supplementary file 1 [file Table1.docx]

**Embase**

Search Strategy:

| Search | Search term |
| --- | --- |
| 1 | exp neoplasm/ or (neoplasm* or cancer or cancers or tumor or tumour or malignan* or oncology).mp. [mp=title, abstract, heading word, drug trade name, original title, device manufacturer, drug manufacturer, device trade name, keyword, floating subheading word] |
| 2 | exp fertility/ or exp reproduction/ or exp reproductive health/ |
| 3 | exp anxiety/ or (worry or worries or concern* or distress or anxiet*).mp. [mp=title, abstract, heading word, drug trade name, original title, device manufacturer, drug manufacturer, device trade name, keyword, floating subheading word] |
| 4 | 2 and 3 |
| 5 | (fertility concern* or reproductive concern* or fertility worr* or reproductive worr* or childbearing concern* or oncofertility concern*).mp. [mp=title, abstract, heading word, drug trade name, original title, device manufacturer, drug manufacturer, device trade name, keyword, floating subheading word] |
| 6 | 4 or 5 |
| 7 | 1 and 6 |

**Cochrane Central Register of Controlled Trials (CENTRAL)**

Platform: The Cochrane Library

Search Strategy:

| Search | Search term |
| --- | --- |
| #1 | (neoplasm* OR cancer OR cancers OR tumor OR tumour OR malignan* OR oncology):ti,ab,kw |
| #2 | (fertility OR reproduction OR "reproductive health"):ti,ab,kw |
| #3 | (worry OR worries OR concern* OR distress OR anxiet*):ti,ab,kw |
| #4 | (#2 AND #3) |
| #5 | ("fertility concern*" OR "reproductive concern*" OR "fertility worr*" OR "reproductive worr*" OR "childbearing concern*" OR "oncofertility concern*"):ti,ab,kw |
| #6 | (#4 OR #5) |
| #7 | (#1 AND #6) |

**Web of Science**

Platform: Clarivate Analytics Web of Science Core Collection
Search Strategy:

| Search | Search term |
| --- | --- |
| #1 | TS=(neoplasm* OR cancer OR cancers OR tumor OR tumour OR malignan* OR oncology) |
| #2 | TS=(fertility OR reproduction OR "reproductive health") |
| #3 | TS=(worry OR worries OR concern* OR distress OR anxiet*) |
| #4 | #2 AND #3 |
| #5 | TS=("fertility concern*" OR "reproductive concern*" OR "fertility worr*" OR "reproductive worr*" OR "childbearing concern*" OR "oncofertility concern*") |
| #6 | #4 OR #5 |
| #7 | #1 AND #6 |

**Scopus**

Platform: Elsevier Scopus
Search Strategy:

( TITLE-ABS-KEY ( neoplasm* OR cancer OR cancers OR tumor OR tumour OR malignan* OR oncology ) ) AND ( ( TITLE-ABS-KEY ( fertility OR reproduction OR "reproductive health" ) AND TITLE-ABS-KEY ( worry OR worries OR concern* OR distress OR anxiet* ) )OR ( TITLE-ABS-KEY ( "fertility concern*" OR "reproductive concern*" OR "fertility worr*" OR "reproductive worr*" OR "childbearing concern*" OR "oncofertility concern*" ) ) )
